# Multi-omics strategies for biomarker discovery and application in personalized oncology

**DOI:** 10.1186/s43556-025-00340-0

**Published:** 2025-11-21

**Authors:** Ziming Jiang, Haoxuan Zhang, Yibo Gao, Yingli Sun

**Affiliations:** 1 Department of Thoracic Surgery, National Cancer Center/National Clinical Research Center for Cancer/Cancer Hospital, Chinese Academy of Medical Sciences and Peking Union Medical College, Beijing, 100021 China; 2 Central Laboratory & Shenzhen Key Laboratory of Epigenetics and Precision Medicine for Cancers, National Cancer Center/National Clinical Research Center for Cancer/Cancer Hospital & Shenzhen Hospital, Chinese Academy of Medical Sciences and Peking Union Medical College, Shenzhen, 518116 China; 3 Laboratory of Translational Medicine, National Cancer Center/National Clinical Research Center for Cancer/Cancer Hospital, Chinese Academy of Medical Sciences and Peking Union Medical College, Beijing, 100021 China; 4 Department of Thoracic Surgery, National Cancer Center/National Clinical Research Center for Cancer/Cancer Hospital & Shenzhen Hospital, Chinese Academy of Medical Sciences and Peking Union Medical College, Shenzhen, 518116 China; 5 Department of Gastroenterology, Shanxi Province Cancer Hospital/Shanxi Hospital Affiliated to Cancers Hospital, Chinese Academy of Medical Sciences/Cancer Hospital Affiliated to Shanxi Medical University, Taiyuan, 030013 China; 6 Key Laboratory of Genomic and Precision Medicine, Beijing Institute of Genomics, Chinese Academy of Sciences, Beijing, 100101 China

**Keywords:** Multi-omics integration, Database, Cancer biomarkers, Personalized medicine, Single-cell and spatial omics

## Abstract

Multi-omics strategies, integrating genomics, transcriptomics, proteomics, and metabolomics, have revolutionized biomarker discovery and enabled novel applications in personalized oncology. Despite rapid technological developments, a comprehensive synthesis addressing integration strategies, analytical workflows, and translational applications has been lacking. This review presents a comprehensive framework of multi-omics integration, encompassing workflows, analytical techniques, and computational tools for both horizontal and vertical integration strategies, with particular emphasis on machine learning and deep learning approaches for data interpretation. Recent applications of multi-omics have yielded promising biomarker panels at the single-molecule, multi-molecule, and cross-omics levels, supporting cancer diagnosis, prognosis, and therapeutic decision-making. However, major challenges persist, particularly in data heterogeneity, reproducibility, and the clinical validation of biomarkers across diverse patient populations. This review also highlights cutting-edge advances in single-cell multi-omics and spatial multi-omics technologies, which are expanding the scope of biomarker discovery and deepening our understanding of tumor heterogeneity. Finally, we discuss the integral role of multi-omics in personalized oncology, with a particular focus on predicting drug responses and optimizing individualized treatment strategies, supported by real-world clinical practice cases. By bridging technological innovations with translational applications, this review aims to provide a valuable resource for researchers and clinicians, offering insights into both current methodologies and future directions for implementing multi-omics data in biomarker discovery and personalized cancer care.

## Introduction

Recent advances in multi-omics technologies have profoundly transformed our understanding of complex biological systems, particularly in cancer research [1–3]. Since the early days of genomics with Sanger sequencing, the field has undergone a rapid evolution through microarray technologies, with the emergence of high-throughput next-generation sequencing (NGS) platforms [4–6]. This progression has expanded into other layers of biological information, including transcriptomics, proteomics, epigenomics, and metabolomics, collectively reflecting the intricate molecular networks that govern cellular life [7]. More recently, the advent of single-cell and spatial multi-omics has enabled unprecedented resolution in characterizing the cellular microenvironment and intercellular communications within tumors, reshaping our insights into cancer biology and therapeutic responses [8–10].

Despite these technological advances, the integration and interpretation of multi-omics data remain significant challenges. The sheer volume, heterogeneity, and complexity of multi-omics datasets, particularly those from single-cell and spatial platforms, necessitate sophisticated computational approaches for meaningful biological inference [11, 12]. Importantly, multi-omics integration offers critical opportunities to elucidate disease mechanisms, discover biomarkers, and develop precision therapeutic strategies [13, 14]. However, the field currently lacks a structured synthesis that systematically connects technological advances with practical workflows and clinical applications. For many researchers, algorithm developers, and clinicians, navigating data processing, intra- and inter-omics integration, and translational implementation remains complex and fragmented. Therefore, a well-organized review is needed to summarize progress, clarify challenges, and highlight opportunities for advancing multi-omics in oncology.

In this review, we focus on three key aspects: (I) a concise overview of actively maintained public multi-omics databases relevant to cancer research; (ii) detailed workflows for multi-omics data processing, quality control, intra-omics harmonization, and cross-omics integration, complemented by cancer-specific case studies; and (iii) a systematic summary of multi-omics-derived biomarkers and their clinical translation challenges. We place particular emphasis on the emerging roles of single-cell and spatial multi-omics, and on how computational strategies such as artificial intelligence and machine learning are reshaping integration approaches and biomarker discovery. Furthermore, we highlight the translational potential of multi-omics biomarkers for predicting drug responses, refining therapeutic regimens, and advancing precision oncology across major cancer types including lung, breast, colorectal, melanoma, and ovarian cancer.

Following the introduction, we first present an overview of available multi-omics data resources, then describe data processing and integration methodologies, followed by a discussion of biomarker discovery and clinical applications in personalized oncology, and finally outline current challenges and future perspectives. This structured approach is intended not only to serve as a reference for researchers but also to provide actionable insights for bridging technological innovations with clinical translation in multi-omics oncology.

## Overview of multi-omics strategies

**Fig. 1:**
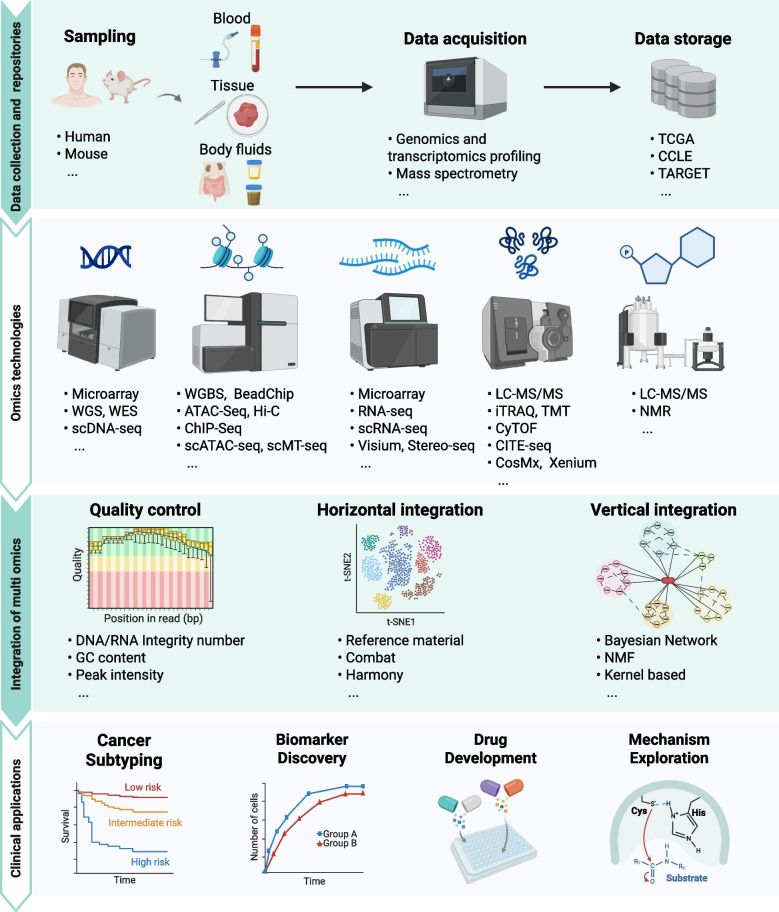
Systematic framework for multi-omics integration. A comprehensive workflow illustrating the multi-layered integration of omics data, encompassing: (1) data acquisition and repositories; (2) implementation and analytical approaches across omics techniques; (3) internal quality control of data, horizontal integration within individual omics layers, and vertical cross-omics integration; and (4) translation into clinical applications

Multi-omics encompasses large-scale, high-throughput analyses of molecular layers including genomics, transcriptomics, proteomics, metabolomics, and epigenomics [11, 15] (Fig. 1). Collectively, these approaches provide a comprehensive understanding of cellular dynamics [16], facilitating biomarker identification that is crucial for cancer diagnosis, prognosis, and therapeutic decision-making. Landmark projects such as The Cancer Genome Atlas (TCGA) Pan-Cancer Atlas, the Pan-Cancer Analysis of Whole Genomes (PCAWG), MSK-IMPACT, and the Clinical Proteomic Tumor Analysis Consortium (CPTAC) have collectively demonstrated the utility of multi-omics in uncovering cancer biology and clinically actionable biomarkers [17–20]. In recent years, multi-omics strategies have become indispensable for biomarker discovery in cancer, enabling the characterization of molecular signatures that drive tumor initiation, progression, and therapeutic resistance [21].

Genomics primarily investigates alterations at the DNA level, leveraging advanced sequencing technologies such as whole exome sequencing (WES) and whole genome sequencing (WGS) to identify copy number variations (CNVs), genetic mutations, and single nucleotide polymorphisms (SNPs) [22]. Genome-wide association studies (GWASs) have been instrumental in identifying cancer-associated genetic variations [22], providing a foundational resource for identifying potential cancer biomarkers. Large-scale sequencing efforts, exemplified by MSK-IMPACT, revealed that approximately 37% of tumors harbor actionable alterations [23]. The tumor mutational burden (TMB), validated in the KEYNOTE-158 trial, has been approved by the FDA as a predictive biomarker for pembrolizumab treatment across solid tumors [24, 25]. These genomic alterations are not only critical for understanding the genetic landscape of cancer but also offer opportunities for precision oncology, where genomic biomarkers guide individualized treatment strategies.

Transcriptomics methods explore RNA expression using probe-based microarrays and next-generation RNA sequencing, encompassing the study of mRNAs, long noncoding RNAs (lncRNAs), miRNAs, and small noncoding RNAs (snRNAs) [26]. The high sensitivity and cost-effectiveness of RNA sequencing have made transcriptomics a dominant component of multi-omics research. Clinically validated gene-expression signatures such as Oncotype DX (21-gene, TAILORx trial) and MammaPrint (70-gene, MINDACT trial) have demonstrated the utility of transcriptomic biomarkers in tailoring adjuvant chemotherapy decisions in patients with breast cancer [27–29].

Proteomics investigates protein abundance, modifications, and interactions using high-throughput methods including reverse-phase protein arrays, liquid chromatography‒mass spectrometry (LC‒MS), and mass spectrometry (MS) [30]. Post-translational modifications such as phosphorylation, acetylation, and ubiquitination represent critical regulatory mechanisms and therapeutic targets [31]. CPTAC studies of ovarian and breast cancers showed that proteomics can be used to identify functional subtypes and reveal potential druggable vulnerabilities missed by genomics alone, directly informing the discovery of protein-based biomarkers for predicting therapeutic responses [32].

Metabolomics examines cellular metabolites, including small molecules, carbohydrates, peptides, lipids, and nucleosides [33]. Techniques like MS, LC‒MS, and gas chromatography‒mass spectrometry enable comprehensive metabolic profiling [34–36]. Classic examples include IDH1/2-mutant gliomas, where the oncometabolite 2-hydroxyglutarate (2-HG) functions as both a diagnostic and a mechanistic biomarker [32]. More recently, a 10-metabolite plasma signature developed in gastric cancer patients demonstrated superior diagnostic accuracy compared with conventional tumor markers [37]. Metabolomics-derived signatures are increasingly recognized as tools for predicting treatment outcomes and tailoring therapeutic strategies.

Epigenomics investigates DNA and histone modifications, including DNA methylation and histone acetylation [38]. Whole genome bisulfite sequencing (WGBS) and ChIP-seq enable comprehensive epigenetic profiling [39]. A classic clinical biomarker of glioblastoma is MGMT promoter methylation, which is a predictor of benefit from temozolomide chemotherapy [40]. Additionally, DNA methylation–based multi-cancer early detection assays (e.g., Galleri test) are under clinical evaluation [41]. Epigenomic alterations therefore serve as both biomarkers and therapeutic targets, with DNMT and HDAC inhibitors already FDA-approved [42, 43].

Recent technological advances have introduced single-cell multi-omics approaches [11, 44], including single-cell genomics, transcriptomics, and proteomics, providing unprecedented resolution in characterizing cellular states and activities [45]. Additionally, spatial transcriptomics and spatial proteomics provide spatially resolved molecular data, enhancing our understanding of tumor heterogeneity and tumor-immune interactions, which are essential for personalized therapeutic strategies in cancer.

In summary, the integration of genomics, transcriptomics, proteomics, metabolomics, and epigenomics provides a multidimensional framework for understanding cancer biology and facilitates the discovery of clinically actionable biomarkers. Additional omics fields, including lipidomics, glycomics, and metagenomics, which are not extensively discussed in this review owing to their limited clinical applications, represent emerging areas with significant potential for future cancer research.

## Resources and approaches for multi-omics data integration

Multi-omics integration involves the comprehensive analysis of omics data from various sources, offering more robust results for biomarker discovery. In this section, we discuss the sources of multi-omics data, the quality control steps, the horizontal integration of intra-omics data, and the vertical integration process of inter-omics data. We also summarize the currently available vertical integration analysis techniques, algorithms, and online tools.

### Data repositories

The exponential growth of multi-omics data, driven by rapid advances in next-generation sequencing technologies, has presented significant challenges in data management [46]. Currently, no unified standard exists for storing and managing multi-omics databases [47]. The organization of multi-omics data varies according to research objectives, cancer types, and temporal characteristics. For instance, single-cell transcriptomics, incorporating cellular dimensional information, requires distinct analytical processes and visualization methods compared with traditional transcriptomics [12, 45]. The increasing complexity and scale of omics data pose substantial challenges for hosting and accessing multi-omics analyses.

Table 1 highlights currently available multi-omics databases that integrate at least two types of omics data. Most of these databases were established for specific research purposes. For example, DriverDBv4 encompasses data from over 70 cancer cohorts, including approximately 24,000 patients, integrating genomic, epigenomic, transcriptomic, and proteomic data [53]. This database employs eight multi-omics integration algorithms to elucidate multi-omics driver characteristics. GliomaDB specifically focuses on glioma research, integrating 21,086 glioblastoma multiforme (GBM) samples from 4,303 patients across multiple platforms including The Cancer Genome Atlas (TCGA), Gene Expression Omnibus (GEO), Chinese Glioma Genome Atlas (CGGA), and Memorial Sloan Kettering-Integrated Mutation Profiling of Actionable Cancer Targets (MSK-IMPACT) [59]. Recently, a comprehensive liver cancer multi-omics database named HCCDBv2 was developed, incorporating clinical phenotype data, bulk transcriptomics, single-cell transcriptomics, and spatial transcriptomics [65]. HCCDBv2 features an intuitive interface facilitating rapid exploration of gene expression patterns across cellular, tissue, and spatial dimensions in liver cancer [65]. Large-scale repositories such as TCGA and TARGET serve as primary sources of publicly available cancer multi-omics data and were established to accommodate extensive cohort datasets [60]. Cross-referencing between repositories has been implemented in various platforms, exemplified by the National Genomics Data Center (NGDC) [49]. This database not only provides access to restricted original data upon application but also incorporates links to public datasets from GEO and TCGA.

**Table 1:** Overview of multi-omics database repositories

Databases	Publication year	Omics types	Cancer types	Sample size	Links	Reference
scCancerExplorer	2023	Single-cell omics (genomics, epigenomics, transcriptomics)	50 cancers	161 single-cell multi-omics datasets, covering over 6.2 million single cells (after quality control)	https://bianlab.cn/scCancerExplorer	[48]
National Genomics Data Center (NGDC)	2024	single-cell, genomics, transcriptomics, epigenomics, metabolomics	various cancers	Raw data: BioProject and BioSample, with 13,487 biological projects and 1,244,954 biological samples collected from 1,549 tissues; Transcriptomics: GEN integrates 34 gene expression datasets from 33 cancer types, covering 2,768 samples; Metabolomics: MACdb integrates 40,710 cancer-metabolite associations from 17 high-incidence or high-mortality cancers, covering 267 features	https://ngdc.cncb.ac.cn/	[49]
MammOnc-DB	2024	Genomics, transcriptomics, epigenomics, proteomics	Breast cancer	Over 20,000 breast cancer samples	http://resource.path.uab.edu/MammOnc-Home.html	[50]
CmirC	2024	Epigenomics, transcriptomics	17 cancers	9,639 samples	https://slsdb.manipal.edu/cmirclust/	[51]
MyeloDB	2024	Genomics, transcriptomics	Multiple myeloma	47 expression profiles, 3 methylation profiles, covering a total of 5,630 patient samples and 25 biomarkers	https://project.iith.ac.in/cgntlab/myelodb/	[52]
DriverDBv4	2024	Genomics, epigenomics, transcriptomics, proteomics	30 + cancers	70 cohorts, approximately 24,000 samples	http://driverdb.bioinfomics.org/	[53]
CoMutDB	2023	Transcriptomics, proteomics	Clear cell renal cell carcinoma (ccRCC)	Data from over 30,000 subjects and 1,747 cancer cell lines	http://www.innovebioinfo.com/Database/CoMutDB/Home.php	[54]
miRDriver	2024	Genomics, epigenomics, transcriptomics	Pan-cancer (18 different cancers)	7,294 patient samples	http://www.mirdriver.org/	[55]
FPIA (Fusion Profiling Interactive Analysis)	2022	Genomics, transcriptomics, proteomics	33 cancers	31,633 fusion events from 6,910 patients	http://bioinfo-sysu.com/fpia	[56]
OncoDB	2022	Genomics, epigenomics, transcriptomics	30 + cancers	Data from over 10,000 cancer patients	http://oncodb.org	[57]
PEN (Protein-Gene Expression Nexus)	2021	Genomics, proteomics	12 cancers	145 cancer cell lines	http://combio.snu.ac.kr/pen	[58]
GliomaDB	2019	Genomics, transcriptomics, epigenomics	Glioma	21,086 samples from 4,303 patients	http://bigd.big.ac.cn/gliomaDB	[59]
TCGA (The Cancer Genome Atlas)	2015	Genomics, transcriptomics, epigenomics	33 cancers	20,000 individual tumor samples	https://www.cancer.gov/ccg/research/genome-sequencing/tcga	[60]
CRI (Cancer Research Institute) iAtlas	2018	Clinical data, genomics, immunology, single-cell transcriptomics	33 cancers	10,000 tumor samples	https://cri-iatlas.org/	[61]
TARGET (Therapeutically Applicable Research to Generate Effective Treatments)	2018	Genomics, transcriptomics	24 pediatric cancers	1,699 pediatric samples	https://www.cancer.gov/ccg/research/genome-sequencing/target	[62]
METABRIC (Molecular Taxonomy of Breast Cancer International Consortium)	2017	Genomics, transcriptomics	Breast cancer	2,503 breast tumor samples	https://ega-archive.org/studies/EGAS00000000083	[63]
TCIA (The Cancer Immunome Database)	2016	Genomics, transcriptomics	20 solid cancers	8,000 tumor samples	https://tcia.at/	[64]

Despite these developments, current databases are not specifically designed for comprehensive multi-omics data integration. This limitation stems from the inherent complexity of multi-omics data, including diverse data sources and challenges in data cleaning and standardization. Additionally, the field lacks standardized protocols for hosting multi-omics data that can effectively address the complexities of various experimental designs.

### Quality control

The integration of multi-omics data enables the transformation from descriptive single-omics snapshots to comprehensive data flow information along the DNA–RNA–protein regulatory cascade, revealing cellular event sequences. However, significant challenges arise in multi-omics data analysis due to biological system complexity and potential technical variations in sample collection, data generation, and analysis processes. Rigorous quality assurance (QA) and quality control (QC) protocols are essential prerequisites for complex multi-omics data processing [66, 67]. According to the International Organization for Standardization (ISO 9000:2015) [68], QA encompasses processes and activities designed to prevent errors and maintain quality standards, whereas QC involves testing and inspection procedures to verify compliance with established quality standards. Quality control standards vary across different omics platforms owing to differences in experimental platforms, manufacturers, sample processing protocols, and sample quality. Each omics field maintains distinct quality control procedures and evaluation metrics [69]. For instance, in proteomics, a typical LC‒MS experiment comprises sample preparation, liquid chromatography, mass spectrometry, and bioinformatics analysis. The process begins with protein digestion into peptides, followed by liquid chromatographic separation and mass spectrometric measurement. Spectral interpretation is then performed through bioinformatics approaches. Proteomics core facilities implement systematic monitoring with defined quality thresholds for each workflow step, including metrics such as peptide–spectrum matches (PSMs), identification rates of peptides and proteins, protein quantity, and sequence coverage. Similarly, single-cell transcriptomics analysis incorporates specific quality metrics, including gene count, unique molecular identifier (UMI) count, mitochondrial proportion, and doublet identification.

Reference materials (RMs) play crucial roles in both QA and QC processes for multi-omics research [67]. RMs are well-characterized samples with known properties that serve multiple purposes: validating analytical method accuracy and reliability, assessing data comparability across laboratories and instruments, and establishing measurement accuracy and precision standards [67, 69]. As exogenous substances introduced at the initiation of omics analysis, RMs effectively correct technical and systematic biases across different sequencing samples [69]. Notable initiatives for establishing omics RMs include the Genome in a Bottle Consortium (GIAB) [70], Microarray/Sequencing Quality Control (MAQC/SEQC) [71, 72], Clinical Proteomic Tumor Analysis Consortium (CPTAC) [73], Metabolomics Quality Assurance and Quality Control Consortium (mQACC) [67], China's Quartet project [69], and EATRIS [74]. However, current omics research faces limitations in standardization, as many omics measurements cannot be traced to the International System of Units (SI units) or associated with physical/chemical property values, unlike DNA/RNA sequencing reads or MS spectra. Additionally, the lack of unified reference material sources hampers the establishment of consistent quality control standards across different datasets.

### Horizontal integration

The initial step of the data integration workflow involves selecting anchor points for alignment, which primarily involves two distinct strategies. The first approach utilizes genomic features as anchors for horizontal integration, and is suitable for datasets of the same omics type with comparable numbers of gene features, such as RNA-seq and microarray datasets [75]. This method preserves most gene features and integrates datasets from a single omics type, aiming to consolidate data across batches, techniques, and laboratories for downstream analysis [75]. Unwanted variations, often referred to as batch effects, can introduce systemic biases and confound critical research factors [76, 77].

A variety of horizontal integration methods have been developed for both bulk and single-cell omics data [76, 78]. For bulk data, after sequencing reads are transformed into normalized values such as fragments per kilobase of transcript per million mapped reads (FPKM) or transcripts per million (TPM) and log-transformed, linear batch correction methods originally designed for bulk datasets (e.g., limma [79] and ComBat [80]) can effectively mitigate biases arising from differences in sequencing depth, sample preparation, or platform-specific variations [76]. These methods ensure compatibility between different RNA-seq datasets and even between RNA-seq and microarray data. However, these approaches inherently assume identical or well-defined cell type compositions between batches and are thus unsuitable for single-cell data.

Horizontal integration methods tailored to single-cell data typically rely on nonlinear or locally linear strategies that account for variations in cell type composition. A range of methods has been developed for batch correction in single-cell data, including mutual nearest neighbors (MNN) [81], Seurat v5 [82], LIGER [83], Harmony [84], and batch balanced K-nearest neighbors (BBKNN) [85]. Seurat employs a MNN algorithm to align data in a joint low-dimensional space defined by principal components or canonical covariates [82]. BBKNN corrects data within a neighborhood graph, offering faster computation at the expense of single-cell resolution [85]. Harmony iteratively learns cell-specific linear correction functions using k-means clustering in a principal component space [84]. Despite their utility, these algorithms sometimes face over-correction issues, which occur when batch correction vectors are incorrectly estimated, forcing mismatched cellular subpopulations to merge. The core challenge lies in distinguishing batch effects from underlying biological signals of interest, particularly when substantial biological variation exists between batches. These methods generally perform well when sequencing platforms, tissue origins, and cell types are consistent.

### Vertical integration

The second strategy for data integration involves using samples or cells as anchors for vertical integration. Vertical integration is applicable to datasets derived from the same sample but assessed through multiple omics techniques, such as genomic sequencing and RNA sequencing from the same tumor tissue, or the integration of bulk RNA-seq with single-cell transcriptomics, and single-cell transcriptomics with spatial transcriptomics [86]. This approach enables the incorporation of biological information from various dimensions, including different layers of the central dogma, cellular characteristics, or spatial data, making vertical integration one of the most valuable strategies in multi-omics research. This finding offers the potential to uncover novel regulatory mechanisms with causal relationships.

Vertical integration strategies leverage explicit correspondences between molecular profiles from matched multi-modal experiments, such as those derived from the same tissue source, individual, or even the same cells or cell populations (e.g., cells collected from the same individual). These correspondences serve as anchors between data modalities, enabling integration across diverse omics datasets. Integration approaches generally fall into three categories [87]. Early integration combines datasets into a unified matrix before constructing a comprehensive model. While this approach ensures simultaneous consideration of all modalities, it typically requires transforming datasets into a common representation, potentially resulting in information loss [88]. The resulting unified matrix often becomes complex and high-dimensional, introducing additional noise. Moreover, in cases of dataset imbalance, features from underrepresented omics layers may receive insufficient consideration. In contrast, late integration develops independent models for each dataset before combining them into a unified framework [89]. Although this approach allows for modality-specific modeling, it often fails to capture critical inter-omics relationships, leading to suboptimal model performance. The inability to adequately represent interactions between omics layers limits both the method's ability to fully utilize multi-modal data and its capacity to elucidate disease mechanisms. Consequently, late integration has not gained widespread adoption in multi-omics research.

Intermediate integration encompasses methods capable of jointly integrating multi-omics datasets without requiring prior transformation or relying on simple concatenation [15]. These approaches typically generate new representations, some common to all omics and others specific to individual omics, enabling subsequent analyses. This step effectively reduces the dimensionality and complexity of multi-omics datasets [90]. Milan Picard et al. further categorized intermediate integration into two subtypes: mixed integration, which involves transforming each omics dataset independently into simpler representations, and intermediate strategies, which integrate multi-omics datasets jointly without requiring prior transformation or relying on concatenation [15]. Overall, intermediate data integration involves constructing a joint model from the datasets. This approach has become the dominant method for handling vertically integrated multi-omics data due to its proven effectiveness in clinical applications such as biomarker discovery and disease subtyping [3]. Notably, intermediate strategies do not necessitate data transformation, thereby avoiding information loss. Table 2 provides a detailed summary of the mainstream algorithms used in intermediate data integration, including their specific applications and available resources.

**Table 2:** Analytical methods for multi-omics integration

Tool	Method category	Omics types	Objectives	Implementation	Publications (Last 10 Years)	Reference
MOFA/MOFA +	FA, JDR	E, G, P, T	DS, MD	R code on GitHub: bioFAM/MOFA	12	[91]
nNMF	NB, JDR	E, T	DS, MD, BD	Not released	4	[92]
intNMF	JDR	E, G, P, T	DS	R package: intNMF	4	[93]
jNMF	JDR	E, T	DS, MD	R code on GitHub: yangzi4/iNMF	2	[94]
JIVE	JDR	E, P, T	DS, MD	R package: r.jive	6	[95]
SLIDE	FA	E, M, P, T	DS, MD, BD	R code on GitHub: irinagain/slide-paper	1	[96]
iCluster	FA, JDR	E, M, P, T	DS, BD	R package: iCluster	28	[97]
iClusterPlus	JDR	E, G, T	DS, BD	R package: iClusterPlus	12	[98]
iClusterBayes	NB, JDR	E, G, T	DS, BD	R package: iClusterPlus	3	[99]
LRAcluster	JDR	G, P, T	DS	R code on: bioinfo.au.tsinghua.edu.cn	3	[100]
NEMO	KB	E, T	DS	R code on GitHub: Shamir-Lab/NEMO	3	[101]
SNF	NB, KB	E, M, P, T	DS	R or MATLAB: compbio.cs.toronto.edu	47	[102]
CIMLR	KB	E, G, T	DS	R or MATLAB on GitHub: danro9685/CIMLR	3	[103]
MixKernel	KB	E, T	DS	R package: mixKernel	1	[104]
FuseNet	NB	G, T	DS	Python package on GitHub: sfu-mial/FuseNet	1	[105]
sPLS-DA	JDR	G, E, P, T	MD	R package: mixOmics	17	[106]
DIABLO	JDR	E, P, T	NA	R package: mixOmics	5	[107]
MCIA	JDR	P, T	DS, MD	R package: omicade4	4	[108]

*Abbreviation: FA* Factor analysis, *JDR* Joint dimensional reduction, *NB* Network-based, *KB* Kernel-based, *E* Epigenomics, *G* Genomics, *P* Proteomics, *T* Transcriptomics, *M* Metabolomics, *DS* Disease subtyping, *MD* Module detection, *BD* Biomarker discovery

Network-based integration methodologies implement sophisticated algorithms to create unified representations from diverse molecular networks. Notable algorithms in this category include similarity network fusion (SNF) [102], FuseNet [105], and iClusterBayes [99]. Among them, SNF has proven particularly effective in recent clinical applications [102]. For instance, Xi and colleagues employed SNF to develop an immune molecular classification (IMC) prognostic system for head and neck squamous cell carcinoma by integrating multi-omics data spanning copy number variations, somatic mutations, DNA methylation, and transcriptomics. They identified one patient group displaying enhanced sensitivity to cisplatin and immunotherapy, and another group demonstrating increased responsiveness to epidermal growth factor receptor (EGFR) inhibitors [109].

Bayesian networks (BNs) represent probabilistic graphical models that synthesize probability theory and graph theory to delineate causal relationships among random variables in biological systems. These models have found extensive applications in systemic biology [110], including protein signaling pathway modeling, gene function prediction, and cellular network inference. The iClusterBayes algorithm exemplifies the successful implementation of Bayesian approaches in multi-omics integration [99]. When applied to TCGA datasets for glioblastoma and renal cancer, iClusterBayes identified distinct genomic patterns through the integration of mutation, copy number alteration, and gene expression [99]. Notably, the survival probability among the identified subtypes demonstrated greater significance compared to classifications based solely on gene expression data [99].

Kernel-based (KB) methods represent a class of statistical machine learning approaches designed for pattern analysis in complex datasets [87]. These methods operate by projecting original data into a higher-dimensional feature space through kernel matrices, enabling sophisticated pattern recognition tasks including clustering, classification, regression, correlation analysis, and feature selection [87]. Notable kernel-based analytical tools encompass support vector machines (SVMs), principal component analysis (PCA), and canonical correlation analysis (CCA) [111].

Factor analysis methods facilitate dimensionality reduction by decomposing datasets into fewer constituent factors. Non-negative matrix factorization (NMF), a widely adopted factor analysis technique, decomposes non-negative data matrices into products of two lower-dimensional non-negative matrices [112]. The application scope of NMF has expanded significantly due to its relationship with k-means clustering, one of the most extensively utilized unsupervised learning algorithms [113]. While traditional NMF addresses homogeneous data clustering, recent developments such as integrative NMF (intNMF) and joint NMF (jNMF) enable heterogeneous data integration [93, 94].

Advanced NMF variants offer distinct advantages in multi-omics integration. Joint NMF identifies modules of correlated multi-omics data through common space analysis [94], whereas intNMF leverages consensus clustering for molecular data integration [93]. A recent innovation, network-based NMF (nNMF), builds upon intNMF by incorporating similarity network fusion (SNF) to integrate consensus matrices from individual omics into a comprehensive network structure for spectral clustering [92]. These intermediate integration methods excel in uncovering joint inter-omics structures while preserving information from different omics datasets with varying feature or sample dimensions. Multi-omics factor analysis (MOFA) has demonstrated practical utility in cancer research [91]. In a study of 116 lung carcinoids, MOFA integrated methylation and gene expression data to identify treatment-relevant molecular subtypes [114]. The analysis revealed five latent factors, with the primary two factors accounting for 45% and 34% of the dataset variance, respectively [114]. Consensus clustering based on these survival-associated factors stratified patients into three distinct clusters with differential survival outcomes and therapeutic targets.

Vertical integration of single-cell omics has emerged as a pivotal focus in multi-omics research, owing to its unprecedented capability to examine biological processes at the single cell level. This integration approach demonstrates significant advantages in understanding cellular heterogeneity and regulatory mechanisms [12]. scRNA-seq enables the inference of cis- or trans-regulatory elements, such as transcription factors or enhancers. The incorporation of ATAC-seq for identifying cis-regulatory elements effectively addresses the challenge of detecting regulatory genes, particularly transcription factors that typically exhibit low abundance in transcriptomic data [115]. Several analytical methods, originally developed for bulk multi-omics analysis, have been successfully adapted for single-cell multimodal data integration. These methods encompass various matrix factorization approaches for unsupervised dimensionality reduction, including MOFA/MOFA+ [116], JIVE [95], partial least squares (PLS) [106], and multiple co-inertia analysis (MCIA) [108]. MOFA and its enhanced version, MOFA+, implement group factor analysis to identify shared variations across multiple modalities [116]. In MOFA+, the observed data in each modality is interpreted as a linear weighted function of an underlying common latent space [116]. This advanced version incorporates multiple underlying latent spaces to account for population effects, such as experimental batch variations. Although not specifically designed for single-cell data, MOFA has demonstrated practical utility in analyzing datasets with joint single-cell methylation and transcriptome profiles [117].

Future developments in this field should focus on two main directions: (1) advancing technological capabilities for simultaneous detection of multiple omics in single cells, as multimodal data can facilitate the development of causal models through comprehensive biological measurements, and (2) developing new causal modeling algorithms specifically designed for single-cell omics that can effectively integrate two or more modalities, thereby enhancing the biological interpretability of multimodal data.

### Online tools and websites

Multi-omics integration involves a range of methodologies tailored to diverse experimental designs and research objectives. Computational strategies for multi-omics integration are generally categorized into knowledge-driven and data-driven approaches [118]. Knowledge-driven methods analyze each omics layer separately, leveraging existing knowledge bases to map identified features. While these methods are straightforward and computationally efficient, their effectiveness is constrained by the quality and comprehensiveness of the reference databases [119, 120]. In contrast, data-driven approaches uncover novel patterns and correlations across omics layers without reliance on prior knowledge [15]. These methods enable the identification of previously unrecognized relationships and provide deeper insights into system-wide interactions [15, 86]. Table 3 presents several online approaches that enable researchers to upload raw data or expression matrices. These methods leverage online web-based tools to facilitate the initial dimensionality reduction of multi-omics data and the construction of regulatory networks.

**Table 3:** Online tools for multi-omics integration

Tool name	Omics type	Input format	Analysis tools	Visualization	Website	Ref
OmicsAnalyst	Transcriptomics, proteomics, metabolomics, microbiome	Matrix	MCIA, CPCA, PLS, DIABLO, SNF, Procrustes analysis, Univariate correlation	Scatter Plot, Dual Heatmap, Correlation Networks	https://www.omicsanalyst.ca/	[121]
MiBiOmics	Transcriptomics, proteomics, metabolomics, microbiome, genomics	Matrix	Univariate correlation, Procrustes analysis, MCIA	Scatter Plot, Dual Heatmap, Correlation Networks	https://shiny-bird.univ-nantes.fr/app/Mibiomics	[122]
3Omics	Transcriptomics, proteomics, metabolomics	Matrix, List	Univariate correlation	Heatmap	https://3omics.cmdm.tw/	[123]
xMWAS	Transcriptomics, proteomics, metabolomics	Matrix	Partial Least Squares (PLS), Sparse PLS, Multilevel Sparse PLS	Networks	https://kuppal.shinyapps.io/xmwas	[124]
PaintOmics 4	Transcriptomics, epigenomics, proteomics, metabolomics	Matrix	Clustering, Correlation analysis	Heatmap, Pathway	https://paintomics.uv.es/	[119]
GraphOmics	Transcriptomics, proteomics, metabolomics	Matrix	Cypher query language, Reactome database mapping	Interactive Pathway Diagram, Interactive Table, pheatmap	https://graphomics.glasgowcompbio.org/	[125]
web-rMKL	Transcriptomics, epigenomics	Text, MAT	Joint dimensionality reduction (rMKL-LPP)	Cluster Assignment, n-dimensional Coordinates (Text Output)	web-rMKL.org	[126]

For comprehensive data understanding, the integration of multiple approaches is strongly recommended whenever feasible. Several data-driven methods, including MCIA, DIABLO, and PLS, facilitate online multi-omics data analysis through dimensionality reduction and network analysis [127]. These joint dimensionality reduction (JDR) methods calculate components that explain major variation trends within the data. Notably, compared with PCA, which is commonly used in single-omics dimensionality reduction, JDR methods can simultaneously compute components across multiple tables. For instance, MCIA identifies components that maximize both variation sources within each dataset and cross-dataset component correlations, thereby capturing shared variation trends across all omics datasets [108, 127].

OmicsAnalyst is a data-driven online platform designed for multi-omics analysis, featuring continuous updates to enhance its functionality [121, 127]. The platform facilitates data-driven integration by leveraging standardized omics data and metadata [121]. The application of these methods requires adherence to specific criteria, including sample size and omics types. For example, OmicsAnalyst mandates a minimum of 20 samples for data-driven integration, along with strict sample matching across different omics layers to ensure reliable analysis [121, 127]. The research team has also developed a comprehensive suite of analytical tools, including ExpressAnalyst for single-transcriptomics and proteomics analyses and MetaboAnalyst for single-lipidomics data processing [128, 129]. These tools support common analytical procedures such as differential expression analysis and functional enrichment. Additionally, knowledge-driven integration can be conducted using OmicsNet [120], which leverages known protein‒protein interactions derived from the STRING database to enhance data interpretation and network analysis.

Another category of widely utilized multi-omics analysis tools includes PaintOmics 4 and GraphOmics [119, 125]. These platforms leverage existing biological knowledge represented in pathway maps to project multi-omics data and visualize them in highly interpretable formats, particularly suitable for metabolomics analysis. PaintOmics 4 enables pathway-based visualization of regulatory relationships across different omics layers, enhancing the interpretability of enrichment analysis [119]. However, this approach faces notable limitations when dealing with non-targeted and semi-targeted metabolomics data due to its dependence on existing databases. GraphOmics, a similar web-based tool relying on the Reactome database, enables users to perform various global analyses, including differential expression and pathway activity analysis. These analyses prioritize differentially expressed molecules based on their alterations under different experimental conditions [125]. Notably, GraphOmics provides an interactive interface for exploring and querying relationships between differentially expressed molecules [125].

In summary, diverse repositories, quality control pipelines, and computational frameworks have been developed to support multi-omics integration, each addressing different challenges in data heterogeneity and complexity. These resources and tools establish a robust methodological foundation for biomarker discovery, enabling the systematic identification and validation of clinically relevant signatures.

## Applications of multi-omics integration in biomarker discovery

Multi-omics technologies have emerged as a primary source of clinical biomarkers due to their capacity for high-throughput, unbiased or targeted detection of diverse biomolecules at scale [130]. In recent decades, continuous advancements have been made in the exploration and discovery of novel, sensitive, specific, and accurate tumor biomarkers. In this section, we summarize multi-omics biomarker development across single-molecule, multi-molecule, and cross-omics integrated biomarker panels. Furthermore, we discuss the major challenges in the development of multi-omics biomarkers.

### Identification of single-parameter biomarkers

Single-molecule biomarkers such as CEA and CA125 have been widely utilized across various cancer types for early screening, prognosis prediction, and recurrence monitoring [131]. These biomarkers have become part of clinical practice due to their historical reliability in indicating the presence of tumors. However, their clinical utility is often limited by insufficient sensitivity, particularly in the early stages of cancer, and their vulnerability to interference from non-cancerous conditions, leading to potential misdiagnosis. For instance, CA125 is commonly used in ovarian cancer, but its sensitivity is suboptimal for early detection, and it may also be elevated in benign conditions such as menstruation, endometriosis, or liver disease [132, 133]. Similarly, CEA, although widely used in colorectal cancer, lacks specificity and is elevated in various non-cancerous diseases, making it unreliable for early-stage diagnosis [134, 135]. Despite these limitations, these traditional biomarkers remain indispensable in the later stages of cancer for monitoring disease progression and recurrence. However, their limitations in early-stage detection and specificity emphasize the need for a broader, more integrated approach to biomarker identification. The advancement of multi-omics technologies has enabled a more comprehensive understanding of regulatory relationships across different levels during specific biological processes or treatment responses in tumors, leading to the identification of novel biomarkers. These biomarkers span multiple molecular levels, including genomic, transcriptomic, epigenomic, proteomic, and metabolomic domains [136, 137].

Notable examples have emerged from large-scale public multi-omics initiatives such as The Cancer Genome Atlas (TCGA) and other extensive sequencing datasets. In lung cancer, somatic mutations in genes including PPP3CA, DOT1L, and FTSJD1 in lung adenocarcinoma, and RASA1 in lung squamous cell carcinoma have been identified as potential drivers of carcinogenesis [138]. Similarly, in esophageal squamous cell carcinoma, mutations in EP300 and CREBBP genes have been recognized as potential oncogenic drivers [139]. In another study, Ziyi Li and colleagues employed single-cell RNA sequencing and spatial transcriptomics to identify POSTN as a key biomarker for predicting immunotherapy response, predominantly expressed by extracellular matrix cancer-associated fibroblasts (EM CAFs) [140]. These fibroblasts were shown to modulate cancer cell reprogramming, epithelial‒mesenchymal transition, and regulatory T cell recruitment, collectively contributing to early recurrence and influencing the efficacy of immunotherapy [141].

**Fig. 2:**
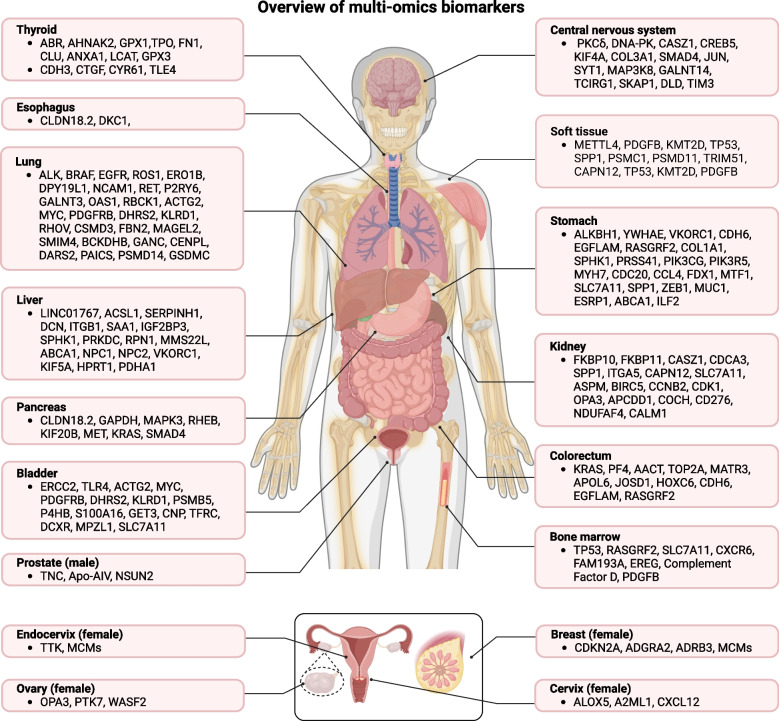
Overview of multi-omics biomarkers. Multi-omics biomarkers identified in various types of cancers over the past five years. The figure highlights key genes and gene families discovered through multi-omics integration

While many biomarker discoveries arise from tissue-based cancer mapping studies, biomarkers derived from more accessible sources such as blood, saliva, urine, ascites, and uterine lavage fluid demonstrate greater potential for clinical application and validation. Additionally, single biomarkers identified through next-generation high-throughput omics typically require validation using simpler, more stable measurement methods, such as qPCR for transcriptomic markers. Nevertheless, the potential for liquid biopsy and multi-omics integration in improving cancer detection, monitoring, and prognosis prediction is enormous. Moving forward, the challenge will be to overcome these clinical barriers, validate these integrated biomarkers in large, diverse patient populations, and develop standardized tools for clinical implementation. This would represent a significant advancement in the quest for more precise, non-invasive, and early-stage cancer diagnostics [142]. Figure 2 provides a comprehensive overview of biomarkers identified across different omics levels in various cancer types.

### Development of multi-gene biomarker panels

The evolution towards multi-gene panels represents an inevitable trend in the omics era, driven by two key factors. First, the development of single-gene biomarkers has reached relative maturity, with diminishing returns in novel marker discovery. Second, advances in omics technologies now enable simultaneous measurement of thousands of genetic features in patient samples. Within these comprehensive profiles, specific alterations in gene expression or protein levels frequently demonstrate strong correlations with tumor characteristics. Furthermore, panels comprising multiple genes often reflect the activation of specific pathways, as exemplified by the co-occurrence of TP53 and KRAS pathway alterations in smoking-associated cancers [143].

The decreasing costs of omics technologies have made multi-gene panel testing economically viable compared to single-gene approaches. A notable example of successful implementation was demonstrated by David Capper and colleagues, who developed a comprehensive DNA methylation profiling system for central nervous system (CNS) tumors [144]. Using the Infinium HumanMethylation450K BeadChip array, they established a reference cohort encompassing 82 distinct CNS tumor classes. Their random forest algorithm-based classification system achieved remarkable accuracy with sensitivity and specificity rates of 0.989 and 0.999, respectively [144].

The subsequent Molecular Neuropathology 2.0 (MNP 2.0) initiative further advanced this approach by integrating DNA methylation analysis, gene panel sequencing, and centralized neuropathological assessment in a population-based pediatric CNS tumor cohort [145]. This comprehensive study revealed that methylation-based classification significantly enhanced diagnostic precision in specific cases. Distinctive correlations emerged between DNA methylation classes and copy number alterations. For instance, the 'infantile hemispheric glioma' methylation class exhibited characteristic focal amplifications at cytoband 2p23.2, indicating ALK gene fusions, whereas the 'PXA' class demonstrated consistent homozygous deletions of the CDKN2A/B locus.

The increasing refinement of molecular disease subtypes and treatment strategies has led to numerous studies developing multi-biomarker panels across various clinical contexts. Examples include exosomal RNA panels for predicting fluoropyrimidine-based neoadjuvant chemotherapy response in advanced gastric cancer, a 21-bacteria probe qPCR panel for immune checkpoint inhibitor response prediction in non-small cell lung cancer, colorectal cancer, and melanoma, and a 10-metabolite GC diagnostic model for early gastric cancer detection and prognosis prediction [146–148].

The Oncotype DX test in breast cancer represents a particularly successful validation of this approach [142]. This 21-gene RT‒PCR assay generates a recurrence score (RS) that predicts disease recurrence probability and identifies patients likely to benefit from adjuvant chemotherapy. Its clinical utility has been validated through multiple clinical trials. For broader biomarker validation, resources such as the UK Biobank and International Cancer Genome Consortium provide unprecedented opportunities for comprehensive evaluation of biomarker panels [143].

### Cross-omics integration for composite biomarker panels

Cross-omics integration offers significant potential for developing composite biomarker panels that improve cancer diagnosis, prognosis, and treatment response prediction. Unlike traditional single-parameter biomarkers, integrating data from multiple omics layers—such as genomics, transcriptomics, proteomics, and metabolomics—provides a more comprehensive view of cancer biology, enhancing both accuracy and robustness in identifying biomarkers. Recent studies in lung cancer have demonstrated that incorporating microRNA and DNA methylation markers, specifically mir-21 and HOXA9 methylation status, into gene expression biomarker panels substantially enhances predictive accuracy compared to single-modality approaches [149]. Despite these advantages, the development of cross-omics biomarker panels faces considerable challenges, including the inherent complexity of high-dimensional data integration and elevated noise levels across different omics platforms. Furthermore, the limited availability of comprehensive cross-omics datasets has constrained research advancements, resulting in few clinically validated applications [1, 150].

Various mathematical frameworks have emerged to facilitate multi-omics data integration, including network-based approaches and matrix factorization methods. Machine learning has become increasingly prominent in this field, yielding promising results. For instance, Hyuk-Jung Kwon and colleagues analyzed blood samples from 92 lung cancer patients and 80 healthy controls, examining cancer markers, cell-free DNA concentrations, and copy number variations [151]. Their machine learning approach, utilizing AdaBoost, Multi-Layer Perceptron, and Logistic Regression algorithms, demonstrated superior diagnostic accuracy compared to single-marker analyses. Similarly, Lin and colleagues developed an integrated multi-omics signature combining whole slide images, cancer-associated fibroblasts, and clinical parameters, achieving enhanced prognostic accuracy for breast invasive ductal carcinoma [152]. One notable initiative, Molecular Neuropathology 2.0 (MNP 2.0), combined DNA methylation and gene sequencing to improve diagnostics in CNS tumors [145]. This approach allowed for the identification of specific genetic alterations, such as ALK gene fusions and CDKN2A/B deletions, which significantly enhanced diagnostic precision [153].

In summary, multi-omics integration has substantially expanded the landscape of biomarker discovery, ranging from traditional single-parameter indicators to sophisticated multi-gene and cross-omics composite panels. Looking ahead, emerging technologies like single-cell sequencing and spatial transcriptomics are expected to further improve cross-omics integration. These techniques allow for the analysis of tumor heterogeneity at unprecedented resolution, offering new insights into cancer biology and therapeutic resistance.

## Challenges and future directions of multi-omics integration in biomarker discovery

Prior to the omics era, numerous biomarkers had already been successfully implemented in clinical practice, such as HER2 for breast cancer and AFP for hepatocellular carcinoma [154, 155]. Lung adenocarcinoma, for instance, could be further classified based on driving mutations in KRAS and/or EGFR genes [156]. The introduction of multi-omics approaches has since reshaped biomarker discovery by providing two transformative advantages: the ability to interrogate a vast array of molecular features in parallel, and the integration of heterogeneous molecular layers to generate composite biomarker panels that capture the complexity of tumor progression and therapeutic response. These strategies offer distinct benefits, including cross-validation of biomarkers across molecular levels to enhance reproducibility and clinical applicability, improvement of diagnostic accuracy through the combination of complementary molecular signatures, and the opportunity to uncover mechanism-based biomarkers by mapping cross-layer molecular interactions. Moreover, signals that are weak or inconsistent in single-omics analyses can be amplified through integrative frameworks, and advanced computational strategies such as artificial intelligence and machine learning are accelerating the identification of novel composite biomarkers with greater predictive potential.

Nevertheless, the development of novel biomarkers or multi-parameter biomarker panels through multi-omics approaches faces two primary limitations. The integration of high-dimensional and heterogeneous datasets increases the risk of false positives due to both statistical overfitting and inherent technical biases, necessitating robust analytical pipelines with stringent control of false discovery rates [157]. Furthermore, the combinatorial complexity generated by integrating multiple data types yields large numbers of candidate biomarkers, each requiring extensive functional validation in experimental systems and independent patient cohorts [158]. These demands place a premium on statistical power, standardization of methodologies, and scalable validation platforms. This section addresses these two major challenges in multi-omics cancer biomarker development and explores future directions.

### Challenges in data integration

The rapid advancement of multi-omics technologies has facilitated the discovery of numerous biomarkers, including those derived from various omics combinations, which has enhanced the development of personalized medicine strategies. However, significant challenges persist in the integration and utilization of multi-omics data.

In biomarker development, different omics features from the same dataset may contribute to marker identification across various regulatory levels. For instance, PDL1 protein expression requires assessment at the protein level, whereas EGFR mutation detection necessitates genomic analysis [159, 160]. The heterogeneity among different omics platforms presents considerable challenges. Each omics technology exhibits distinct precision levels, and signal‒to‒noise ratios significantly impact data integration. Furthermore, the nature of data varies substantially—transcriptomics generates continuous measurements, while genomic features such as CNV, SNP, or methylation often produce discrete values. The disparity in feature quantities among different omics layers influences their relative weights during integration, necessitating careful consideration in weighting strategies [11].

The handling of missing values poses a significant challenge in multi-omics data integration. Certain features may be undetectable in some samples, particularly in proteomics and metabolomics analyses [161, 162]. In cohort studies, complete multi-omics data collection for all individuals is often unfeasible, resulting in substantially smaller complete-case sample sizes compared to the total cohort. While algorithms such as MOFA can facilitate sample subgroup identification, data imputation, and outlier detection, imputation methods may compromise dataset reliability and generate data structures that violate independence assumptions required by many statistical frameworks [91]. High-quality multi-omics datasets, such as TCGA and emerging single-cell or spatial transcriptomics projects, may provide new opportunities for biomarker development [163].

### Verification of biomarkers

The development and clinical validation of cancer biomarkers have faced significant challenges over the past three decades, resulting in limited successful translation of novel biomarkers into clinical practice. Although the clinical value of biomarkers stems from their predictive capabilities and ability to discriminate disease classifications, biomarker development often originates from establishing multi-omics reference atlases, leading to observational and empirical characteristics in biomarker research [164]. Investigators frequently lack clarity regarding the scope and types of data collection at study initiation, quality control protocols for data inclusion/exclusion, timing of data analysis, and decisions about additional data collection following preliminary analyses. The complexity of omics data exacerbates these challenges, as the number of identified features substantially exceeds the sample size, significantly increasing the likelihood of false-positive results [165]. This challenge persists even during omics marker validation within the same cohort, whether at the same level or across different molecular levels (such as RNA and protein), and more critically, these findings often fail to replicate in independent datasets [165].

The discovery of tissue-based biomarkers presents additional limitations. While many studies initially obtain tissue samples through biopsies and tumor resections, the invasive nature of these procedures complicates subsequent biopsies for independent cohort validation and monitoring treatment response or tumor recurrence across multiple time points. Furthermore, tumor heterogeneity, characterized by multiple malignant cell clones, may prevent single biopsies from accurately representing the entire tumor landscape [166]. The development of biomarker detection methods urgently requires optimization for clinical applications, particularly those utilizing less invasive sampling approaches (blood, saliva, and urine) [167]. However, clinical validation faces substantial obstacles due to the extended timeframes, increased costs, and difficulties in obtaining high-quality samples for external validation required for most biomarker optimization methods [166].

As multi-omics models increasingly incorporate multiple parameters as predictive indicators, establishing robust computational frameworks becomes crucial, particularly for machine learning-based biomarker models. A critical issue in multi-parameter modeling is overfitting, which typically occurs when numerous potential predictors are used to differentiate a limited number of outcome events [168]. Biomarker panels that demonstrate excellent predictive performance within the same cohort may fail to generalize to other cohorts. Therefore, multi-parameter prediction models require early determination of discovery cohort size, quality standards, and validation criteria, which should be consistently applied to external validation in non-overlapping patient cohorts. The classification thresholds and model adjustment stringency should be predetermined to minimize artificial effects from overfitting. Additionally, internal validation of discovery cohorts (cross-validation) serves to calibrate predictor selection stringency and reduce features to a small, robust core set, where the absence of any element would significantly diminish predictive power. Notably, overfitting is particularly prevalent in single-cell omics datasets due to cohort size limitations. Moreover, the non-linear nature and black-box characteristics of machine learning algorithms, such as deep neural networks, enable them to highly fit subtle patterns and even noise in training data. When using these algorithms, it is essential to provide substantial training data volume, avoid excessive training parameters and extended training periods, and prioritize simpler, more transparent models, such as linear or generalized linear models.

In summary, while multi-omics integration has already reshaped biomarker discovery, substantial challenges remain in data harmonization, clinical validation, and model generalizability. Nevertheless, it is undeniable that multi-omics biomarkers are gradually advancing toward clinical translation in personalized oncology. In the following section, we will discuss the emerging applications of multi-omics biomarkers in clinical practice and their potential to enhance precision oncology strategies.

## Applications for multi-omics biomarkers in personalized oncology

**Fig. 3:**
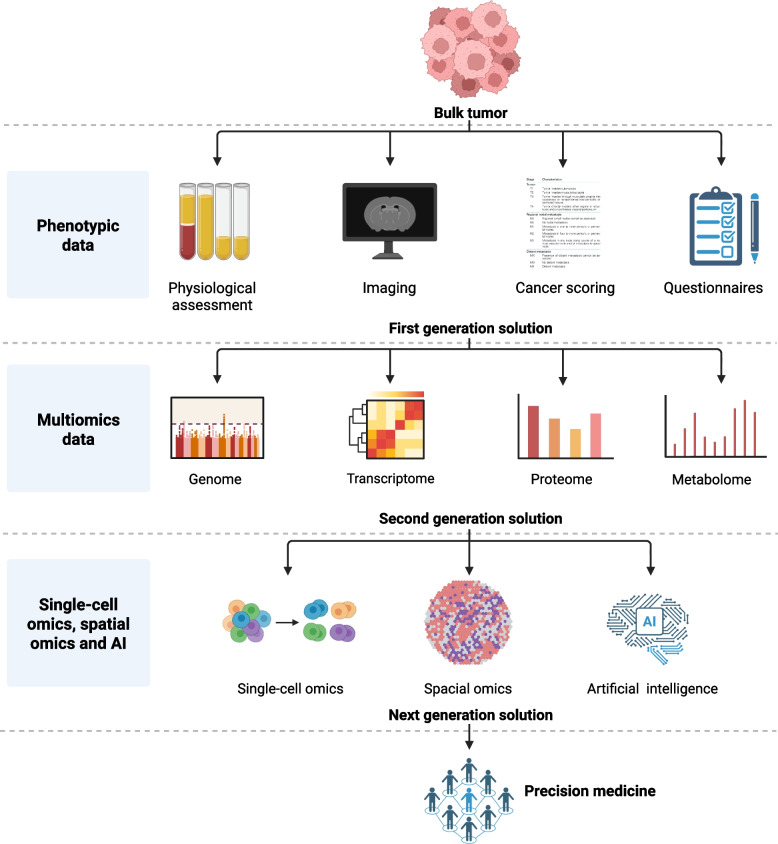
Multi-omics strategies for personalized medicine in cancers. Three generations of personalized medicine solutions are presented, and artificial intelligence is considered crucial for integrating multi-omics data to enable personalized medicine

Translating multi-omics biomarkers into clinical decision-making represents the next critical step toward realizing the promise of personalized medicine. Although significant challenges remain, recent advances demonstrate that robust biomarker development can effectively inform individualized therapeutic strategies. For instance, patient-derived organoids integrated with comprehensive omics profiling enable personalized drug screening tailored to tumor-specific features [169]. More broadly, personalized medicine, which tailors treatment and prevention strategies to an individual's genetic, environmental, and lifestyle characteristics [11], has transformed cancer care from the traditional “one-size-fits-all” paradigm to delivering the right therapy to the right patient at the right dose and time [170]. This approach relies on biomarker-driven patient stratification to maximize therapeutic benefit [171]. Multi-omics technologies have established a powerful foundation by integrating molecular and clinical data into diagnostic, prognostic, and therapeutic frameworks (Fig. 3).

As previously discussed, biomarker discovery has evolved from single-gene markers to comprehensive molecular signatures derived from genomics, transcriptomics, proteomics, metabolomics, single-cell multi-omics [172] and spatial multi-omics [173]. Coupled with advances in artificial intelligence and machine learning, these strategies enable the extraction of clinically actionable features from high-dimensional datasets. Once validated, biomarkers such as circulating tumor DNA, immune-related gene signatures, and metabolite profiles will be pivotal in guiding individualized therapeutic decisions, thereby solidifying the role of multi-omics in advancing personalized medicine.

### Prediction of drug responses

The application of multi-omics biomarkers in personalized treatment is gradually becoming a significant research focus in personalized medicine. By integrating genomic, transcriptomic, epigenomic, proteomic, and metabolomic data, researchers can reveal disease mechanisms, predict therapeutic responses, and develop novel biomarkers, advancing personalized medicine in drug treatments. Table 4 summarizes cases of multi-omics approaches and biomarkers used in the prediction of drug responses.

**Table 4:** Multi-omics and biomarkers in prediction of drug responses

Tumor Type	Multi-Omics Strategies	Biomarkers	Drugs	Predictive Type	References
Breast Cancer	Genomics, Transcriptomics, Epigenomics	HSD17B4 methylation	HER2-targeted drugs	HSD17B4 methylation silencing as a predictive biomarker for HER2-positive breast cancer treated with HER2-targeted therapy	[174]
	Genomics, Epigenomics, Proteomics	DNA methylation at enhancer CpGs	Neoadjuvant chemotherapy and bevacizumab	Epigenetic explanation and prediction of response to neoadjuvant chemotherapy and bevacizumab in breast cancer	[175]
Hepatocellular Crcinoma	Genomics, Epigenomics, Transcriptomics, Proteomics	Biomarker biobank associated with drug responses	mTOR inhibitor Temsirolimus and multikinase inhibitor Lenvatinib	Establishing a patient-derived liver cancer organoid biobank (LICOB) for prognosis-related subtype identification and drug screening	[176]
	Transcriptomics, Proteomics, Lipidomics, Metabolomics	FAD subtypes	Anti-PD-1 therapy, Sorafenib, TACE	Molecular classification of HCC based on the fatty acid degradation (FAD) pathway for personalized treatment	[177]
	Genomics, Transcriptomics, Proteomics, Phosphoproteomics	HCC proteomic subtypes	Sorafenib	dentifying HCC subtypes with distinct clinical outcomes and discovering nine proteins related to metabolic reprogramming as potential subtype-specific biomarkers	[178]
Melanoma	Genomics, Transcriptomics, Immunomics	Multi-modal predictor of response	Ipilimumab, Nivolumab	Multi-omics prediction of melanoma response to immune checkpoint blockade	[179]
Colorectal Cancer	Multi-omics data	TAPBP	PD-1 blockade and COX inhibitors	Study of PD-1 blockade combined with COX inhibitors in dMMR metastatic colorectal cancer	[180]
	Genomics, Transcriptomics, Immunomics	G2M checkpoint pathway and MYC pathway	Regorafenib, Nivolumab	Multi-omics analysis of tumors in MSS/pMMR metastatic colorectal cancer patients treated with Regorafenib plus Nivolumab (REGONIVO) or TAS-116 plus Nivolumab (TASNIVO)	[181]
	Histopathology, Genomics, Transcriptomics, Single-cell Omics	CRLM PDO	5-FU or FOLFIRI chemotherapy regimens	Organoid biobank of 50 patients with colorectal liver metastases (CRLM) analyzed for inter- and intra-patient heterogeneity	[182]
	Genomics, Epigenomics, Transcriptomics, Clinical data	t-RNA aminoacylation	Standard and non-standard drugs	Multi-omics analysis of PDOs for drug sensitivity prediction in advanced colorectal cancer	[183]
Ovarian Cancer	Single-cell Omics	Drug-resistance subtypes	First-line chemotherapy	AI in drug resistance in ovarian cancer: subtype classification and prognosis modeling	[184]

Firstly, multi-omics biomarkers demonstrate broad potential across various cancer types. In HER2-positive breast cancer, integrating genomic, transcriptomic, and epigenomic data revealed that methylation of the HSD17B4 gene as a biomarker predicts sensitivity to HER2-targeted therapies, providing new insights for improving treatment precision [174]. Similarly, another study combined genomic, epigenomic, and proteomic data to demonstrate how epigenetic events explain and predict responses to neoadjuvant chemotherapy and bevacizumab in breast cancer, offering new perspectives on treatment selection [175]. In hepatocellular carcinoma (HCC), multi-omics approaches have identified molecular features related to prognosis and therapeutic response. Studies have integrated genomic, transcriptomic, proteomic, and metabolomic data to classify HCC molecular subtypes based on fatty acid degradation (FAD) associated biomarkers. These classifications have been used to evaluate targeted therapies like sorafenib for personalized treatment [177, 178]. In melanoma, multi-omics integration of tumor and immune cell data enables the prediction of responses to immune checkpoint blockade, providing a foundation for precision treatment and a reference for designing immunotherapy strategies for other immune-related tumors [179]. Colorectal cancer (CRC) studies have also advanced drug sensitivity prediction and therapeutic optimization using multi-omics. For example, multi-omics analysis showed TAPBP may serve as a biomarker for immune checkpoint inhibitor therapy to predict responses of combining PD-1 blockade with COX inhibitors in patients with metastatic CRC [180]. Furthermore, comprehensive analysis of MSS/pMMR metastatic CRC tumors treated with regorafenib plus nivolumab (REGONIVO) or TAS-116 plus nivolumab (TASNIVO) has helped identify biomarkers for therapeutic efficacy [181].

Second, organoid models, particularly patient-derived organoids (PDOs), offer a physiologically relevant platform for multi-omics analyses in cancer research. In HCC, a liver cancer organoid biobank (LICOB) has enabled genomic, epigenomic, proteomic, and metabolomic data integration to reveal response patterns to mTOR inhibitors and multi-target tyrosine kinase inhibitors through biomarker features associated with drug responses [176]. In CRC, PDO models have been used to predict drug sensitivity through multi-omics analysis, exploring the efficacy of standard and non-standard therapies [182]. Similarly, PDOs derived from CRC liver metastases have captured intrapatient and interpatient heterogeneity, aiding chemotherapy predictions [183]. 

Moreover, artificial intelligence (AI), particularly deep learning models, provides powerful tools for processing and integrating multi-omics data. AI demonstrates extensive potential in data dimensionality reduction, feature extraction, and predictive modeling, enabling rapid and accurate predictions for clinical decision-making. For complex cancers like CRC and ovarian cancer, AI algorithms have facilitated multi-omics data analysis and biomarker identification, uncovering factors related to drug sensitivity and resistance [182, 184].

By combining multi-omics technologies and biomarkers with innovative AI methods, personalized treatment research is entering a new phase. Leveraging comprehensive data analysis allows deeper insights into tumor mechanisms, optimizes therapeutic strategies, and improves patient outcomes. The integration of organoid models, multi-omics techniques, and AI approaches will continue to drive clinical translation in personalized medicine, laying a solid foundation for achieving the goals of personalized medicine.

### Optimization of treatment plans

In optimizing tumor treatment, multi-omics technologies and biomarkers are playing an increasingly important role. Multi-omics integration strategies have revealed the molecular characteristics and biomarkers of various tumor types, offering new perspectives for personalized treatment (Table 5).

**Table 5:** Multi-omics in optimization of cancer treatment plans

Cancer type	Multi-omics strategies	Biomarkers	Treatment optimization method	Reference
Gastric Cancer	Genomics, Transcriptomics, Single-Cell Omics, Spatial Omics	DCN	Multi-omics analysis reveals CAFs in the tumor microenvironment and identifies DCN as a representative marker of dCAF and a potential negative predictor of ICB response	[185]
	Genomics, Single-Cell Omics, Immunomics	Pyroptosis risk score	Predicts the effect of neoadjuvant immunotherapy through pyroptosis risk score (PRS); low PRS is associated with enhanced anti-tumor immune cell infiltration	[186]
	Transcriptomics, Epigenomics	Cancer subtypes	Multi-omics data identify three subtypes associated with different clinical outcomes, and mutations, feature gene sets, driver genes, and chemotherapy sensitivity are identified for each subtype	[187]
	Multi-Omics Analysis	EMT pathway	Establishing stable gastric cancer cell lines (SPDO1P and SPDO1LM) to analyze their multi-omics features to predict drug sensitivity and provide a basis for personalized treatment	[188]
Hepatocellular Carcinoma	Genomics, Transcriptomics, Lipidomics, Metabolomics, Proteomics, Single-Cell Omics	FAD subtypes	Molecular classification via fatty acid degradation (FAD) pathway to provide personalized treatment strategies for HCC patients	[177]
	Multi-Omics Analysis of Mitochondrial Cell Death-Related Genes	Mitochondrial cell death index	Predicts prognosis and clinical translation of hepatocellular carcinoma (LIHC) through mitochondrial cell death index (MCDI); MCDI correlates with immune infiltration, TIDE score, and sorafenib sensitivity	[189]
Lung Cancer	SARS-CoV-2-Related Gene Multi-Omics Analysis	SARS-CoV-2 score	Multi-omics analysis reveals the impact of SARS-CoV-2 infection on prognosis, immune microenvironment, and treatment strategies in lung adenocarcinoma, providing guidance for personalized treatment	[190]
	Circulating Immune Analysis, Gene Expression Analysis, Gut Microbiome Analysis	Immune cell subtypes	Multi-omics analysis identifies immune cell subgroups and gene expression levels related to progression-free survival (PFS), offering predictions for PD-L1 < 50% NSCLC patients receiving first-line pembrolizumab therapy	[191]
	Multi-Omics Analysis	Tissue resident memory T cells (Trm) infiltration	Multi-omics analysis reveals different response mechanisms of primary lung adenocarcinoma to neoadjuvant immunotherapy, providing a basis for personalized treatment	[192]
Breast Cancer	Genomics, Transcriptomics, Proteomics	Breast cancer subtypes	Integrating copy number variations, gene expression, and protein interaction networks from 73 basal breast cancer samples to propose optimal combination treatment plans for each patient	[193]
Chronic Myelogenous Leukemia	Single-Cell Multi-Omics Analysis	Hematopoietic stem cells (HSCs) subtypes	Single-cell multi-omics analysis reveals the relationship between treatment response and cell heterogeneity in CML patients, providing guidance for personalized treatment	[194]

Multi-omics biomarkers are being used in studying the diversity of gastric cancer and its microenvironment to improve the treatment. Through single-cell RNA sequencing and spatial transcriptomics analysis, researchers have revealed the critical role of the dCAF subtype in cancer-associated fibroblasts (CAFs) in resistance to immune checkpoint inhibitors (ICBs), identifying the representative marker DCN as a potential negative predictive biomarker [185]. Additionally, the pyroptosis risk score (PRS) has been used to predict the effectiveness of neoadjuvant immunotherapy, with findings showing that patients with a low PRS are associated with enhanced anti-tumor immune cell infiltration [186]. Li et al. performed integrated analysis of mRNA, microRNA, and DNA methylation, classifying gastric cancer into three subtypes, each with distinct mutation features and chemotherapy sensitivities [187]. Similarly, for metastatic gastric cancer, Yang et al. established stable cell lines through multi-omics analysis and identified the EMT pathway as a biomarker, which helped predict drug sensitivity and guide personalized therapy [188].

In the molecular subtyping and treatment response of hepatocellular carcinoma (HCC), multi-omics studies have further divided HCC into different subtypes. Through the fatty acid degradation (FAD) pathways, the immune suppressive microenvironment characteristics were revealed, and response capabilities to sorafenib and anti-PD-1 treatments were predicted [177]. Additionally, through multi-omics analysis of mitochondrial-related genes, a mitochondrial cell death index (MCDI) was established to provide a basis for prognosis prediction and treatment guidance [189].

In lung cancer, multi-omics and biomarkers strategies have also made forward-looking contributions to treatment optimization. For lung adenocarcinoma, multi-omics research revealed the effects of SARS-CoV-2 infection and SARS-CoV-2 score (Cov-2S) as a biomarker on the immune microenvironment and treatment strategies, offering new insights for therapeutic decision-making [190]. For non-small cell lung cancer (NSCLC), a multi-omics analysis combining circulating immune and gut microbiome data identified key factors affecting progression-free survival (PFS), optimizing first-line therapy for PD-L1 low-expression patients [191]. Moreover, for triple-negative breast cancer, the use of liquid biopsy and machine learning algorithms significantly improved the precision of personalized treatment [193]. Similarly, single-cell multi-omics analysis of chronic myelogenous leukemia (CML) revealed the connection between treatment response and cell heterogeneity, advancing personalized treatment strategies [194].

**Fig. 4:**
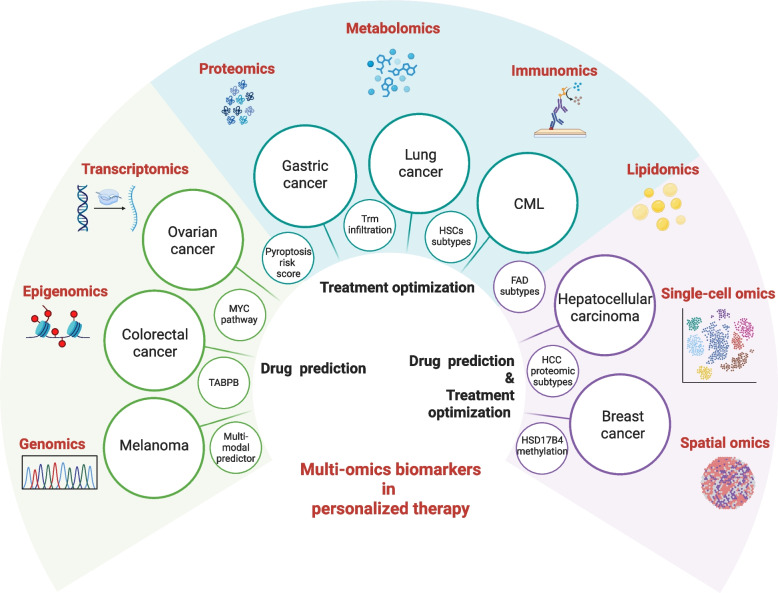
Multi-omics biomarkers in personalized therapy. Multi-omics biomarkers are utilized in drug responses prediction and optimization of cancer treatment plans. Genomics, epigenomics, transcriptomics, proteomics, metabolomics, immunomics, lipidomics, single-cell omics and spatial omics are the most commonly applied multi-omics strategies in personalized cancer therapy

In summary, the optimization of tumor treatment through multi-omics technologies is continuously revealing new molecular mechanisms and predictive biomarkers. These studies not only enhance our understanding of tumor heterogeneity but also provide strong support for the development of precision treatment plans (Fig. 4).

### Clinical practice cases and outcomes

**Fig. 5:**
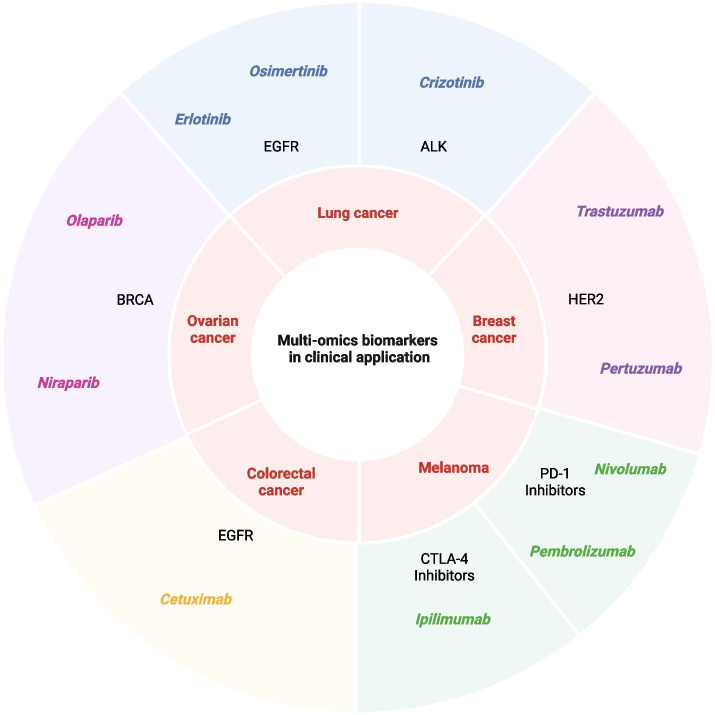
Multi-omics biomarkers in clinical practice of cancer. The figure highlights the major genes as biomarkers for tumor treatment driven by multi-omics strategies and the corresponding targeted drugs

Multi-omics strategies have been confirmed to be effective in the clinical application of precision tumor treatment. Figure 5 illustrates successful cases of multi-omics strategies applied to cancer treatment in clinical practice. Targeted therapy for EGFR and ALK mutations in lung cancer is one of the most successful applications of multi-omics strategies in cancer treatment. Lung cancer, especially NSCLC, is one of the most common malignancies. Multi-omics strategies combining genomics and transcriptomics data have helped identify key markers of EGFR mutations and ALK gene fusions, providing precise evidence for targeted therapy [195]. In recent years, precision treatment for lung cancer has gradually been applied in clinical settings, particularly in patients with EGFR mutations and ALK gene fusions. Targeted drugs like erlotinib and crizotinib have shown excellent clinical efficacy [196]. Studies have shown that patients with EGFR mutations respond well to targeted drugs (such as erlotinib), significantly increasing PFS in patients with EGFR mutations [197]. Subsequent third-generation EGFR-TKI osimertinib overcame resistance to first-generation TKIs, especially targeting the T790M mutation, a common resistance mechanism after EGFR-TKI therapy. Osimertinib showed superior PFS and more favorable toxicity profiles in advanced NSCLC patients with EGFR mutations compared to erlotinib or gefitinib [198]. Moreover, multi-omics data has demonstrated significant efficacy of ALK-targeted drugs (such as crizotinib) in ALK-positive lung cancer patients, improving overall survival [199], highlighting the powerful role of multi-omics strategies in precision therapy for patients with lung cancer.

In breast cancer, multi-omics strategies have led to breakthrough advances in targeted therapy for HER2-positive patients. By integrating genomics, proteomics, and transcriptomics, multi-omics approaches have helped more accurately identify HER2-positive patients and guide personalized treatment [200, 201]. Targeted therapies such as trastuzumab (Herceptin) and pertuzumab (Perjeta) have shown significant efficacy in HER2-positive breast cancer patients, with improvements in both PFS and OS [202]. Additionally, multi-omics data suggest that changes in HER2 expression levels are correlated with therapeutic outcomes. Through integrated genomics and transcriptomics analysis, different subtypes of HER2-positive breast cancer were found to respond differently to treatments, providing more guidance for clinical therapy [203].

In melanoma, immune checkpoint inhibitors such as PD-1 inhibitors and CTLA-4 inhibitors have become crucial treatment options. Multi-omics strategies have provided essential information for predicting immune therapy efficacy and assessing the immune microenvironment [204]. In melanoma treatment, integrating multi-omics has enhanced the clinical efficacy of immune checkpoint inhibitors (ICIs), such as PD-1 inhibitors (nivolumab, pembrolizumab) and CTLA-4 inhibitors (ipilimumab). Recent studies have explored the role of tumor mutational burden (TMB) and neoantigen analysis as predictive biomarkers for PD-1 inhibitor responses. High TMB and the presence of specific neoantigens in tumors have been linked to improved prognosis in melanoma patients receiving ICI therapy [205]. Additionally, the combination of genomic data and immune cell analysis has been shown to identify melanoma patients more likely to benefit from combination PD-1 and CTLA-4 inhibitor therapy [206]. These studies highlight the importance of multi-omics in not only predicting treatment response but also identifying novel biomarkers to improve melanoma treatment outcomes.

In colorectal cancer, targeted therapies (such as EGFR-targeted antibody therapy) and immune therapies (such as PD-1 inhibitors) have become widely used. Multi-omics strategies have helped guide personalized treatment by analyzing tumor mutational burden and immune cell infiltration [207]. For example, EGFR inhibitors like cetuximab have shown significant efficacy in colorectal cancer patients without KRAS mutations, extending progression-free survival [208]. Similarly, colorectal cancer patients with high tumor mutational burden (TMB) respond better to PD-1 inhibitor therapy [209].

In ovarian cancer, multi-omics strategies have facilitated the clinical use of PARP inhibitors. PARP inhibitors such as olaparib and niraparib have become essential targeted therapies, particularly for patients with BRCA gene mutations, and are now included in clinical guidelines [210]. Recent studies integrating genomics, transcriptomics, and proteomics have found that the therapeutic effects of PARP inhibitors are linked to specific molecular features, providing valuable guidance for personalized medicine [211, 212]. Clinical research has shown a strong correlation between BRCA gene mutations and PARP inhibitors: ovarian cancer patients with BRCA1/2 mutations respond well to PARP inhibitors, which inhibit DNA repair through a "synthetic lethality" mechanism, significantly extending PFS [213]. The combined analysis of genomics and transcriptomics has helped identify changes in BRCA mutations and other DNA repair-related genes, further optimizing the clinical application of PARP inhibitors [214]. PARP inhibitors have been shown to significantly extend survival in BRCA-mutated ovarian cancer patients, especially in first-line therapy and maintenance therapy after recurrence [215].

This review highlights how multi-omics biomarkers are reshaping personalized oncology by enhancing drug response prediction, refining treatment optimization, and supporting clinical translation across diverse cancer types. These advances underscore the transformative potential of integrating multi-omics approaches with machine learning, patient-derived models, and innovative clinical strategies to achieve truly individualized care. Nevertheless, despite these promising developments, significant challenges remain that must be addressed before multi-omics biomarkers can be fully and reliably implemented in clinical practice.

## Challenges in the clinical translation of multi-omics biomarkers

The widespread adoption of molecular analysis in cancer patients for precision therapy represents a promising direction in cancer treatment. While many successes have been achieved, it also faces significant practical challenges. Among these, tumor heterogeneity and the integration of tumor molecular subtypes with clinical data stand out as major obstacles, but they also present great potential. In this section, we summarize the key difficulties and possible development directions for applying multi-omics strategies in cancer precision therapy.

### Patient heterogeneity

As cancer progresses, the accumulation of somatic mutations leads to a rich genetic diversity, resulting in genetically distinct cancer cell subclones, which forms the basis of tumor heterogeneity [216]. The heterogeneity of these cancer cell subclones contributes to tumor resistance and poor prognosis, making a single biopsy sample potentially inadequate to represent the tumor's biological state [217, 218]. Comprehensive tumor sampling aids in evaluating intra-tumor heterogeneity, but this usually requires multiple regions from surgically resected specimens, which imposes a significant economic burden and is not always feasible [166]. Moreover, tumors evolve over time, with gene expression and mutation spectra potentially undergoing dynamic changes, which challenges the stability of therapeutic targets [219]. Additionally, the TME, consisting of immune cells, stromal cells, and blood vessels, also impacts treatment efficacy, and the dynamic changes in the TME add complexity to research and application [220]. In this context, single-cell omics technologies have emerged as a possible solution. Single-cell sequencing technology allows precise capture of genomic, transcriptomic, and epigenomic features of individual cells within limited specimens, helping to elucidate the diversity and dynamic changes of tumor cell types [2]. Furthermore, when combined with spatial transcriptomics, single-cell omics can further reveal the spatial heterogeneity of the tumor microenvironment, potentially offering new solutions for applying multi-omics in cancer precision therapy [221].

### Integration of clinical data

Another significant barrier to the successful application of multi-omics in clinical cancer therapy is the integration of clinical data. Clinical multi-omics data are complex and diverse: different omics data (e.g., genomics, transcriptomics, proteomics, and metabolomics) come from different sources, are massive in scale, and present difficulties in standardization and integration analysis. Real-world data often lack completeness, as patients' medical histories, treatment responses, and imaging data may not be fully digitized or standardized, increasing the difficulty of integration [222]. Moreover, there is a gap between biological and clinical information—how to link molecular subtyping results with specific clinical decisions (e.g., drug selection) still requires further research and validation [223]. Therefore, a series of measures are needed to promote the integration of multi-omics with traditional clinical data. Standardization of laboratory and testing technologies, prospective clinical validation, and clinical feasibility regarding testing time, economic cost, and regulatory aspects are considered key requirements [168]. Additionally, the development of cross-omics analysis tools, such as machine learning and AI algorithms, has made it possible to integrate multi-omics data, for example, by using feature selection methods to identify important molecular markers [224]. Establishing multi-center databases through international cooperation to create standardized multi-omics and clinical databases helps eliminate biases in data sources and promote the application of personalized medicine [225]. To date, numerous initiatives have been launched to promote the integration of molecular and clinical data to enable personalized clinical decision-making and precision therapy [226, 227], and these efforts will continue to contribute to the clinical application of multi-omics data.

In summary, patient heterogeneity and the complexity of clinical data integration remain key barriers to the clinical translation of multi-omics biomarkers. While single-cell and spatial omics technologies, along with machine learning–based integration frameworks, offer promising solutions, their clinical utility requires further validation and standardization. Overcoming these challenges is crucial to ensure reproducibility and scalability, paving the way for future advances in personalized oncology.

## Conclusion and discussion

In this review, we systematically explored the integration of multi-omics technologies for cancer biomarker discovery and their applications in personalized oncology. We provided a structured framework addressing data collection, preprocessing, quality control, and both horizontal (within the same omics type) and vertical (across different omics modalities) integration. This framework aims to simplify the complexity of multi-omics data and facilitate actionable insights. We systematically evaluated publicly available databases, algorithms, and tools, verifying their accessibility and offering direct sources for various integration strategies. Given that these resources may not be universally applicable, we compiled detailed metadata for each database, including omics type, cancer specificity, and sample size. For integrative algorithms, we additionally noted compatible omics layers and practical examples. This structured overview facilitates the selection of appropriate workflows tailored to specific research needs, ultimately enhancing the robustness and reproducibility of multi-omics integration studies.

We also highlight current multi-omics applications in biomarker identification and clinical translation, offering valuable insights for clinicians and translational researchers. Beyond traditional single-gene markers, multi-gene and cross-omics biomarker panels have demonstrated superior sensitivity and specificity, enabling the prediction of therapeutic responses and the optimization of treatment regimens. Patient-derived organoid models, in combination with machine learning, are increasingly facilitating individualized drug screening, while emerging single-cell and spatial omics approaches provide higher-resolution insights into tumor biology and the tumor microenvironment. These advances underscore the transformative potential of multi-omics in guiding precision oncology.

Nevertheless, substantial challenges remain. Barriers such as data standardization, reproducibility, cross-population validation, and the integration of biomarker findings into clinical workflows continue to limit the routine use of multi-omics biomarkers. This review also has limitations: rapid technological developments mean that some emerging methods may not be fully captured, and the inherent complexity of multi-omics datasets complicates harmonization and reproducibility. Furthermore, while representative clinical applications have been discussed, larger and more diverse patient cohorts are needed to confirm their clinical utility.

Future efforts should focus on overcoming integration and standardization challenges through international collaboration, open-source databases, and standardized protocols. Continued development of analytical tools tailored to single-cell and spatial technologies, alongside rigorous clinical validation and adoption of AI-driven approaches, will significantly advance the clinical application of multi-omics technologies, ultimately enabling truly personalized cancer care.

## Data Availability

Not applicable.
